# Identification and Validation of New DNA-PKcs Inhibitors through High-Throughput Virtual Screening and Experimental Verification

**DOI:** 10.3390/ijms25147982

**Published:** 2024-07-22

**Authors:** Liujiang Dai, Pengfei Yu, Hongjie Fan, Wei Xia, Yaopeng Zhao, Pengfei Zhang, John Z. H. Zhang, Haiping Zhang, Yang Chen

**Affiliations:** 1 Department of Physiology, Guangxi University of Chinese Medicine, Nanning 530200, China; 2 Guangdong Immune Cell Therapy Engineering and Technology Research Center, Center for Protein and Cell-Based Drugs, Institute of Biomedicine and Biotechnology, Shenzhen Institutes of Advanced Technology, Chinese Academy of Sciences, Shenzhen 518055, China; 3 Ganjiang Chinese Medicine Innovation Center, Nanchang 330000, China; 4 Faculty of Synthetic Biology and Institute of Synthetic Biology, Shenzhen Institutes of Advanced Technology, Chinese Academy of Sciences, Shenzhen 518055, China; 5 CAS Key Laboratory of Separation Science for Analytical Chemistry, Dalian Institute of Chemical Physics, Chinese Academy of Sciences, Dalian 116023, China; 6 Guangdong Key Laboratory of Nanomedicine, CAS-HK Joint Lab of Biomaterials, CAS Key Laboratory of Biomedical Imaging Science and System, Shenzhen Engineering Laboratory of Nanomedicine and Nanoformulations, CAS Key Lab for Health Informatics, Shenzhen Institutes of Advanced Technology, Chinese Academy of Sciences, Shenzhen 518055, China

**Keywords:** DNA-PKcs, deep learning, virtual screening, CRISPR/Cas9, HDR, anticancer activity

## Abstract

DNA-PKcs is a crucial protein target involved in DNA repair and response pathways, with its abnormal activity closely associated with the occurrence and progression of various cancers. In this study, we employed a deep learning-based screening and molecular dynamics (MD) simulation-based pipeline, identifying eight candidates for DNA-PKcs targets. Subsequent experiments revealed the effective inhibition of DNA-PKcs-mediated cell proliferation by three small molecules (5025-0002, M769-1095, and V008-1080). These molecules exhibited anticancer activity with IC_50_ (inhibitory concentration at 50%) values of 152.6 μM, 30.71 μM, and 74.84 μM, respectively. Notably, V008-1080 enhanced homology-directed repair (HDR) mediated by CRISPR/Cas9 while inhibiting non-homologous end joining (NHEJ) efficiency. Further investigations into the structure-activity relationships unveiled the binding sites and critical interactions between these small molecules and DNA-PKcs. This is the first application of DeepBindGCN_RG in a real drug screening task, and the successful discovery of a novel DNA-PKcs inhibitor demonstrates its efficiency as a core component in the screening pipeline. Moreover, this study provides important insights for exploring novel anticancer therapeutics and advancing the development of gene editing techniques by targeting DNA-PKcs.

## 1. Introduction

DNA-PK (DNA-dependent protein kinase) is a nuclear serine/threonine protein kinase composed of a large catalytic subunit (DNA-PKcs) and a regulatory heterodimer DNA-targeting subunit Ku [1,2,3]. It plays an important role in maintaining genome stability during double-strand breaks (DSBs). Eukaryotic cells respond to DSB by initiating two major repair mechanisms, including canonical non-homologous end-joining (NHEJ) and homology-directed repair (HDR). In the absence of DNA homology donors, cells repair DSBs via the NHEJ pathway [4,5]. Otherwise, cells initiate the HDR pathway with the homology donors. DNA-PK is a major component of the NHEJ pathway: first, the Ku protein recruits DNA-PKcs to the DSB site; DNA-PKcs then recruits and phosphorylates Artemis (a nuclease); finally, Artemis processes the DNA ends before ligation with XRCC4/ligase IV complex [6].

Cells deficient in DNA-PK, being unable to respond to DNA lesions, are hypersensitive to ionizing radiation and radio-mimetic drugs [7]. Such cells eventually undergo cell-cycle arrest and subsequent cell death. Conversely, improper response to DSBs due to the deregulated DNA-PK activity leads to the accumulation of non-lethal DNA damage, genomic instability, and potentially carcinogenic events. The hyperactivation of DNA-PK is associated with many cancers, including melanoma, hepatocellular carcinoma, and multiple myeloma. Consequently, DNA-PK has emerged as a therapeutic target in malignancies [8]. Conventional cancer treatments, including radiotherapies and chemotherapies, can be compromised by hyperactive DNA-PK activity. The inhibition of DNA-PK (including DNA-PKcs) by small molecules like M3814 and NU7441 can sensitize cancer cells to ionizing radiation and chemotherapeutic agents [9,10,11,12].

In addition to combating cancers, inhibiting DNA-PK, including DNA-PKcs, can facilitate HDR-mediated knock-in by reducing the NHEJ events after DSBs induced by nucleases, such as I-Sce1 meganuclease, Zinc finger nucleases, TALENs, or CRISPR-Cas systems [13,14,15,16,17]. For example, M3814, NU7441, and AZD7648 have achieved a higher efficient integration of exogenous donors via the HDR mechanism compared to control groups [18,19,20]. However, most of the DNA-PKcs inhibitors have been metabolically unstable with poor pharmacokinetic properties [21,22]. A few small molecules like M3814 and AZD7648 that avoided such defects had been developed for clinical trials in cancer treatment [10,23]. Currently, there are no FDA-approved DNA-PK inhibitors. Thus, it is of great significance to discover more potent and selective DNA-PKcs inhibitors.

In recent years, virtual screening, a computational drug discovery technique, has been developed to identify potential drug candidates by screening large databases of compounds. It can rapidly screen thousands to millions of compounds, significantly reducing the cost of drug discovery and experimental failures. Unlike traditional virtual screening methods that rely on simplified representations and predefined features of protein structures and ligands, deep learning-based models use neural networks to learn directly from raw input data like the molecular structures of proteins and ligands. We previously developed novel deep learning-based models like DeepBindBC and DeepBindGCN_BC by including non-binding protein–ligand interactions [24,25]. These models are well-suited for large-scale screening tasks and are significantly faster than structure-dependent methods [26].

Molecular dynamics (MD) simulations have also become an important technique in the drug discovery pipeline. MD simulations allow for the exploration of the dynamic behavior and interactions of biomolecules at an atomic level, providing valuable insights into the atomic details of binding poses and the stability of protein–ligand complexes. MD simulations can capture the subtle conformational changes and dynamic interactions occurring between proteins and ligands [27,28]. The development of sampling techniques like metadynamics further helps explore the free energy landscape of protein–ligand binding. We previously developed a pocket MD strategy that greatly facilitates MD simulation.

In this study, we integrated MD simulations into the deep learning-based drug screening pipeline to identify novel small molecules targeting DNA-PKcs with potent anti-tumor activity, as well as high HDR promotion activity at the expense of NHEJ. The deep learning-based methods DeepBindGCN_BC and DeepBindGCN_RG were utilized for fast lead identification, followed by docking and pocket MD simulation. We identified eight compounds for experimental validation, with one displaying remarkable specific anti-tumor activity and HDR promotion activity. We also gained insights into the binding mechanisms and assessed the stability of the resulting complexes from the docking and MD simulation. This information was utilized to prioritize potential drug candidates and select those with favorable binding properties for further experimental validation.

## 2. Results

### 2.1. Computational Methods Aid DNA-PKcs Inhibitor Screening

We developed two deep learning methods, DeepBindGCN_RG and DeepBindGCN_BC, to predict protein–ligand interaction. Additionally, we proposed a pocket molecular dynamics (pMD) simulation in our previous work [25]. In this study, we incorporated DeepBindGCN_BC, DeepBindGCN_RG, Schrödinger docking, pocket MD, and metadynamics to efficiently identify potential inhibitors of DNA-PKcs. Those final selected potential inhibitors were then subjected to further experimental validation.

**Figure 1:**
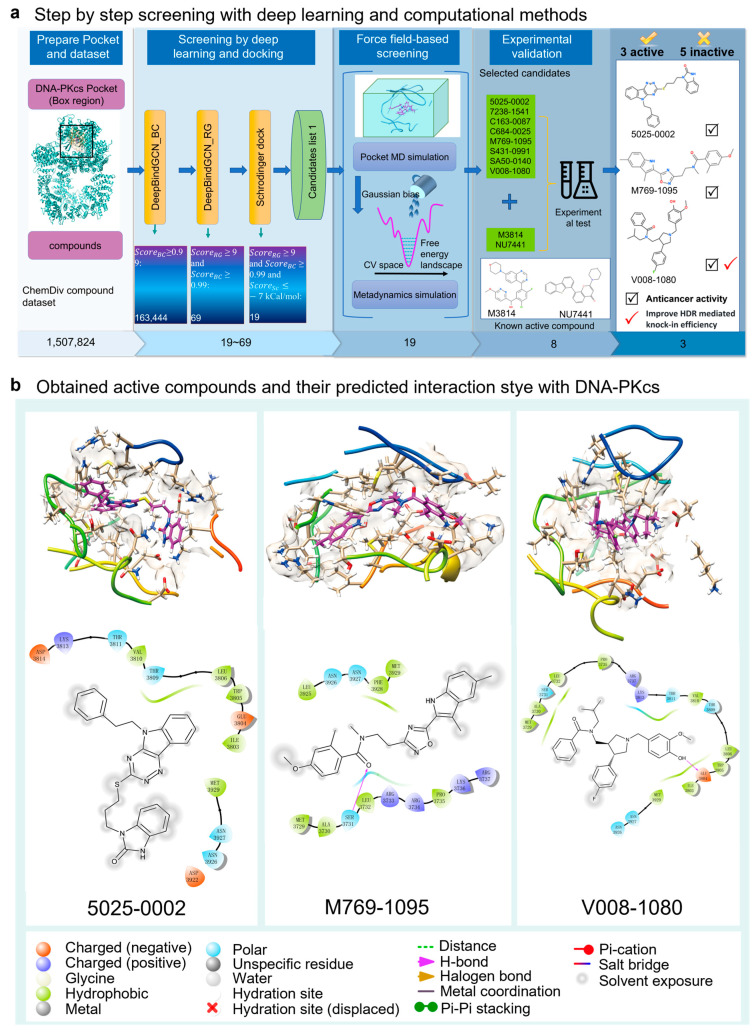
The virtual screening procedure integrates DeepBindGCN models with other methods to identify highly reliable drug candidates for DNA-PKcs. (**a**) The screening inhibitors against the Chemdiv dataset for DNA-PKcs using a combination of DeepBindGCN_BC/RG, Schrödinger docking, MD simulation, and experimental methods. (**b**) The last frame from the 40 ns pocket MD simulation of the three identified active compounds, showing both 3D and 2D interaction details.

The screening workflow consists of four main parts: pocket and dataset preparation, deep learning and docking-based screening, force field-based screening, and experimental validation (Figure 1). In the deep learning-based screening stage, DeepBindGCN_BC was used initially due to its fast speed and ability to exclude non-binder compounds. Subsequently, DeepBindGCN_RG was employed to efficiently identify strong binders. By applying score cutoff values of 0.99 for DeepBindGCN_BC and 9.0 for DeepBindGCN_RG, the initial candidate pool was narrowed down to 69 compounds. After deep learning and docking screening, we obtain 19 candidates with a score cutoff value of DeepBindGCN_BC ≥ 0.99, DeepBindGCN_RG ≥ 9, and Schrödinger ≤ −7 Kcal/mol (Table 1). The cutoff values of 0.99 for DeepBindGCN_BC and 9.0 for DeepBindGCN_RG were chosen based on our experience and the feasibility experimental test. We acknowledge that alternative cutoffs, such as 0.995 for DeepBindGCN_BC and 0.85 for DeepBindGCN_RG, could also be effective. We recommend that users remain flexible in selecting cutoff values based on their specific research needs.

**Figure 2:**
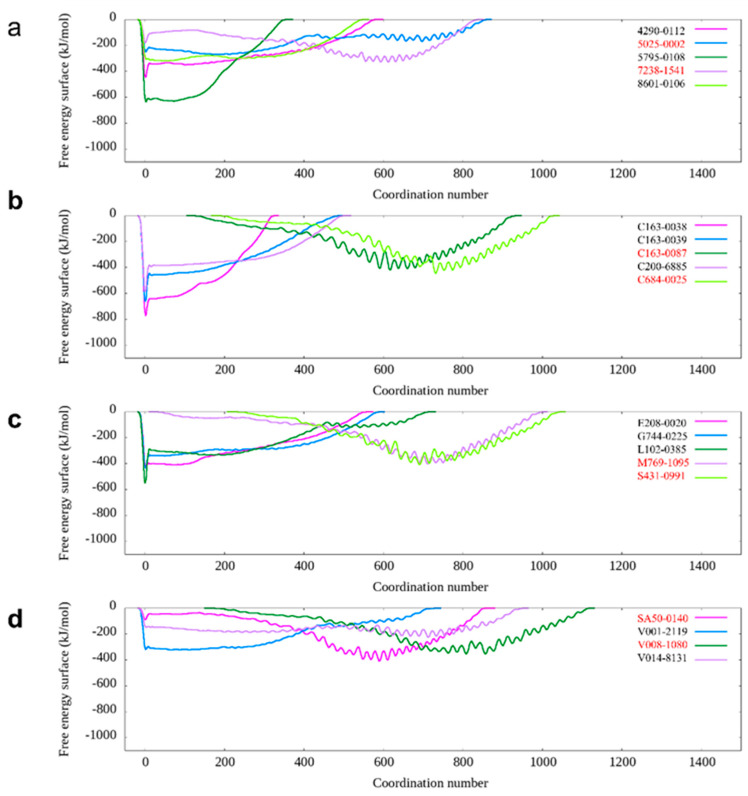
The calculated free energy landscape from metadynamics simulation for those candidates with favorable binding with DNA-PKcs. (**a**) The calculated free energy landscape for DNA-PKcs with candidates 4290-0112, 5025-0002, 5795-0108, 7238-1541, and 8601-0106. (**b**) The calculated free energy landscape for DNA-PKcs with candidates C163-0038, C163-0039, C163-0087, C200-6885, and C684-0025. (**c**) The calculated free energy landscape for DNA-PKcs with candidates E208-0020, G744-0225, L102-0385, M769-1095 and S431-0991. (**d**) The calculated free energy landscape for DNA-PKcs with candidates SA50-0140, V001-2119, V008-1080 and V014-8131.

**Figure 3:**
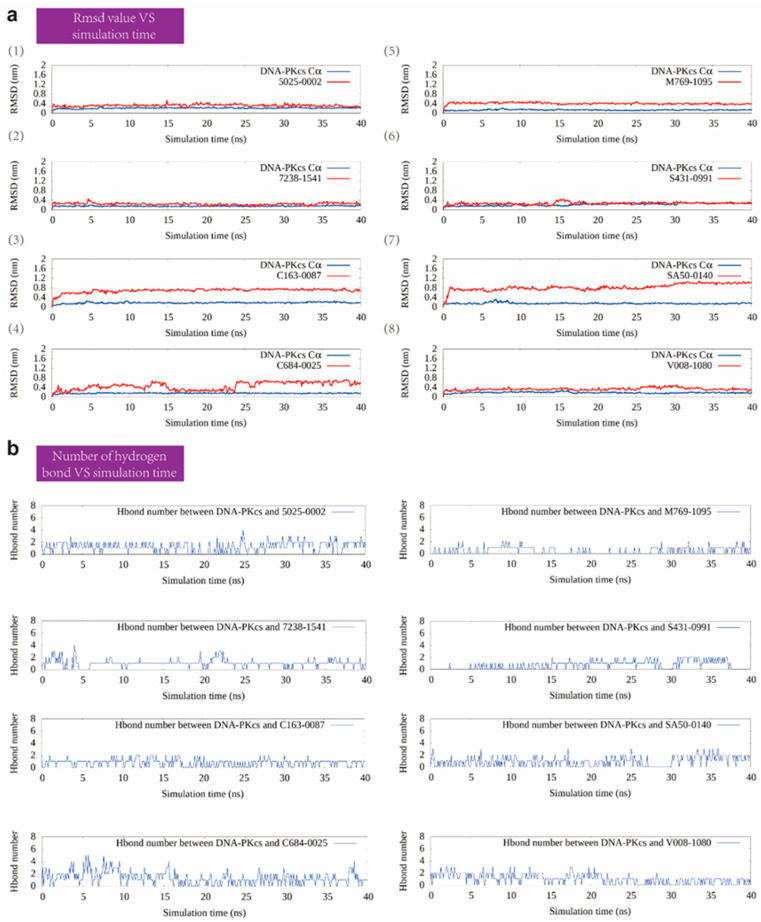
The RMSD value and number of hydrogen bonds of selected compounds with DNA-PKcs during the MD simulation. (**a**) The RMSD value over the 40 ns MD simulation for the eight selected protein–compound complexes. (**b**) The number of hydrogen bonds between DNA-PKcs and the eight selected compounds.

In the subsequent screening stage, pMD simulation, and metadynamics simulation were utilized. Finally, eight compounds were selected for experimental testing, and their chemical structures were listed (Supplementary Figure S1). These compounds were primarily selected based on the predicted free energy landscapes obtained from the metadynamics simulation (Figure 2). The simulations revealed that all eight selected compounds (marked in red) exhibited their lowest energy basin at a coordination number significantly greater than zero. A further analysis of the RMSD values for the eight compounds during the 40 ns pMD simulation indicated that most of the ligands exhibited relatively low and stable deviations from their initial conformations (Figure 3). This suggests that the ligands maintained structural stability throughout the simulation. We also analyzed the RMSF (root mean square fluctuation) of pocket residues and ligands during the pMD simulation. The final selected eight compounds and their corresponding neighboring residues exhibited relatively small fluctuations, indicating stable binding, as shown in Supplementary Figure S2. The residue IDs shown have been reindexed (Supplementary Figure S2), with their corresponding original residue IDs in PDB 7OTY available (Supplementary Table S2). Overall, the RMSD analysis and free energy landscape predictions suggest that most ligands demonstrated stability and favorable binding characteristics during the simulation.

### 2.2. Effects of Small Molecules on 786-O Cell Viability and Proliferation Inhibition

DNA-PKcs is overexpressed in 786-O human Renal cell carcinoma (RCC) cell lines [29]. Small molecules which exhibit anti-proliferation capacity or cytotoxicity against the cell line may be potential DNA-PKcs inhibitors. The potential DNA-PKcs inhibition effects of the eight candidates in 786-O human RCC cells were investigated. All the compounds, along with three representative DNA-PKcs inhibitors (M3814, NU7026, and NU7441) and other DNA-PK inhibitors (KU-0060648 and LY294002) were tested using a CCK8 assay.

**Figure 4:**
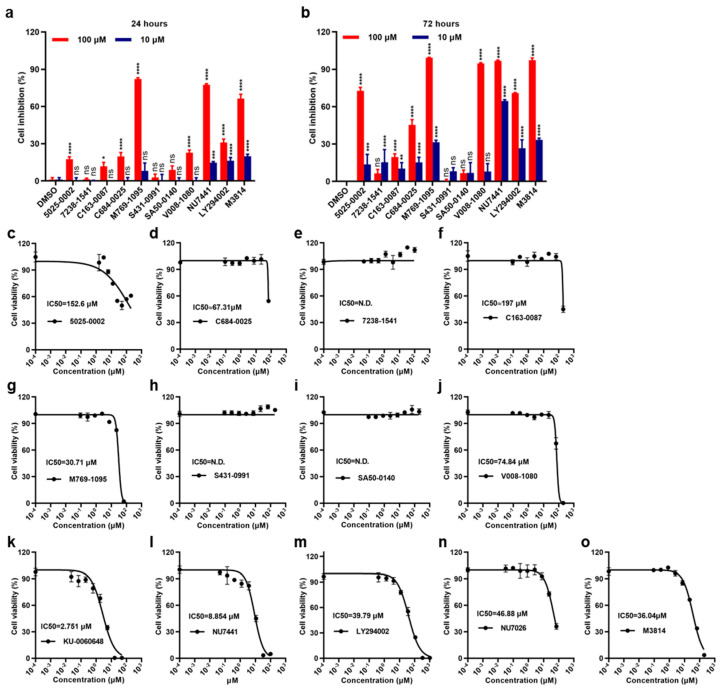
DNA-PKcs inhibitors induce proliferation inhibition in 786-O RCC cells. (**a**,**b**) 786-O RCC cells were treated with M3814, other small molecules (10 μM, 100 μM) or vehicle control (0.1% DMSO) for applied time; cell inhibition was analyzed by CCK8 assay. (**c**–**o**) the cell viability IC_50_ values of different small molecules against 786-O cells. All *p*-values were obtained by comparing to the DMSO group at the same concentration. ns denotes not significant, * denotes *p* < 0.05, ** denotes *p* < 0.01, *** denotes *p* < 0.001, **** denotes *p* < 0.0001.

The results showed that these small molecules displayed different cytotoxicities against the 786-O cell line. Compounds 7238-1541, C163-0087, C684-0025, S431-0991 and SA50-0140 did not show remarkable anti-proliferation effects. In contrast, 5025-0002, M769-1095, and V008-1080 strongly inhibited 786-0 RCC cell growth and induced cell death, especially after 72 h (Figure 4a,b). Furthermore, the cell viability IC_50_ values of these compounds against the 786-O cells were investigated (Figure 4c–j). The average IC_50_ values were 152.6 μM for 5025-0002, 30.71 μM for M769-1095, and 74.84 μM for V008-1080. All the known DNA-PK inhibitors, including DNA-PKcs inhibitors, exhibited excellent cytotoxicity against the 786-O cell line (Figure 4k–o). Additionally, the DNA-PKcs inhibition effects of some representative small molecules were tested in non-cancerous proximal tubule epithelial HK-2 cells, a control cell line with low expression of DNA-PKcs. The results showed M769-1095 and V008-1080 did not exhibit cytotoxicity against HK-2 cells (Supplementary Figure S3). Together, these results demonstrated that some of the screened molecules displayed cytotoxicities against the 786-O RCC cells.

### 2.3. Increase in HDR-Mediated Knock-In by CRISPR/Cas9

**Figure 5:**
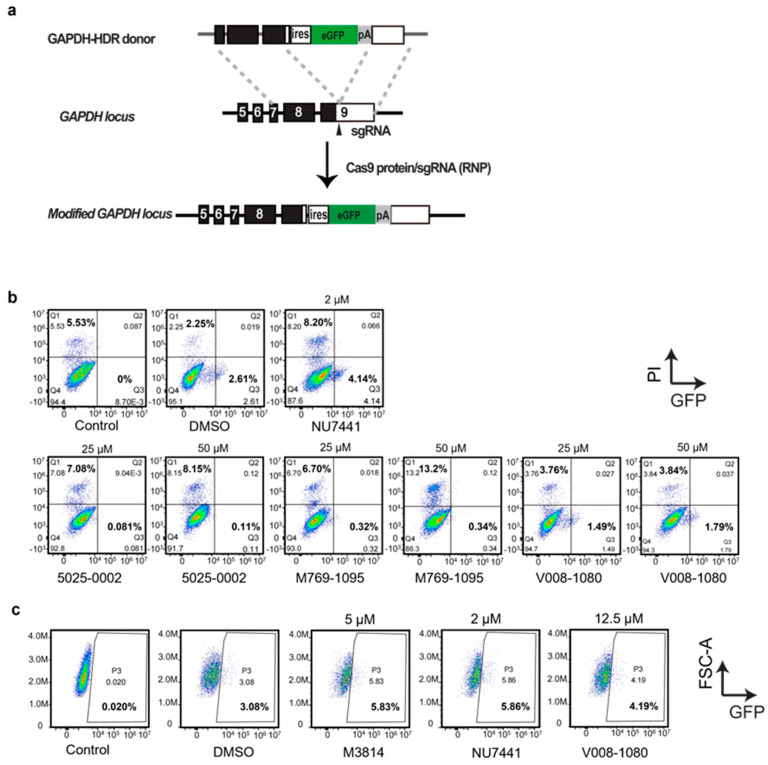
Evaluation of HDR-mediated gene targeting efficiency by treatment with different small molecules. (**a**) Schematics of the donor plasmid and targeting strategy for HDR-mediated knock-in of the ires-GFP reporter at GAPDH 3-UTR. (**b**,**c**) FACS analysis of HEK293T cells treated with different small molecules or vehicles showing HDR-mediated integration of ires-GFP in the presence of RNP mixture and ires-GFP donor. The cells were co-transfected with donor/RNP by nucleofection and analyzed two days post transfection.

DNA-PKcs inhibitors not only display anti-tumor activities but also facilitate HDR-mediated knock-in after DSB by inhibiting the NHEJ pathway. Next, we investigated whether these screened molecules possessed the activities. We applied an established reporter system targeting the GAPDH locus in the human genome [30]. The sgRNA was designed to target GAPDH 3′-UTR near the coding sequences (CDS). A donor carrying a promoterless IRES-eGFP sequence, flanked by two homology arms (HAs) was constructed (Figure 5a). The Cas9 protein–gRNA complex (RNP), combined with the donor, were nucleofected into HEK293T cells. The nucleofected cells were treated with 5025-0002, M769-1095, or V008-1080 at concentrations of 25 μM and 50 μM. GFP protein expression allowed for a direct assessment of the HDR-mediated knock-in efficiency by FACS analysis. At 50 μM, 5025-0002 and M769-1095 induced significant cell death detected by propidium iodide (PI) staining (8.15% and 13.2%) and decreased HDR efficiency (0.11% and 0.34%) (Figure 5b). This high concentration likely induced severe cytotoxicity, masking the actual gene editing efficiency. Increasing the concentration of 5025-0002 and M769-1095 from 25 μM (PI positive, 7.08% and 6.70%) to 50 μM (PI positive, 8.15% and 13.2%) resulted in a higher percentage of dead cells (Figure 5b), indicating non-specific cytotoxicity against HEK293T cells at these concentrations. V008-1080 exhibited weaker cytotoxicity against HEK293T cells (3.76% at 25 μM, 3.84% at 50 μM), although the increased HDR-mediated knock-in efficiency by V008-1080 compared to DMSO (2.61%) was not seen at concentrations of 25 μM (1.49%) and 50 μM (1.79%) (Figure 5b). A slight increase in HDR-mediated knock-in was found when the concentration of V008-1080 was adjusted to 12.5 μM (4.19%, compared to DMSO group 3.08%) (Figure 5c), likely due to a balance between cytotoxicity and the HDR promotion effect of V008-1080. Similarly, when the concentration was adjusted to 10 μM, NU7441 did not show any HDR promotion effect (1.70%, compared to DMSO group 3.25%) (Supplementary Figure S4). However, 5025-0002 or M769-1095 still did not show any HDR promotion activity when the concentration was reduced.

**Figure 6:**
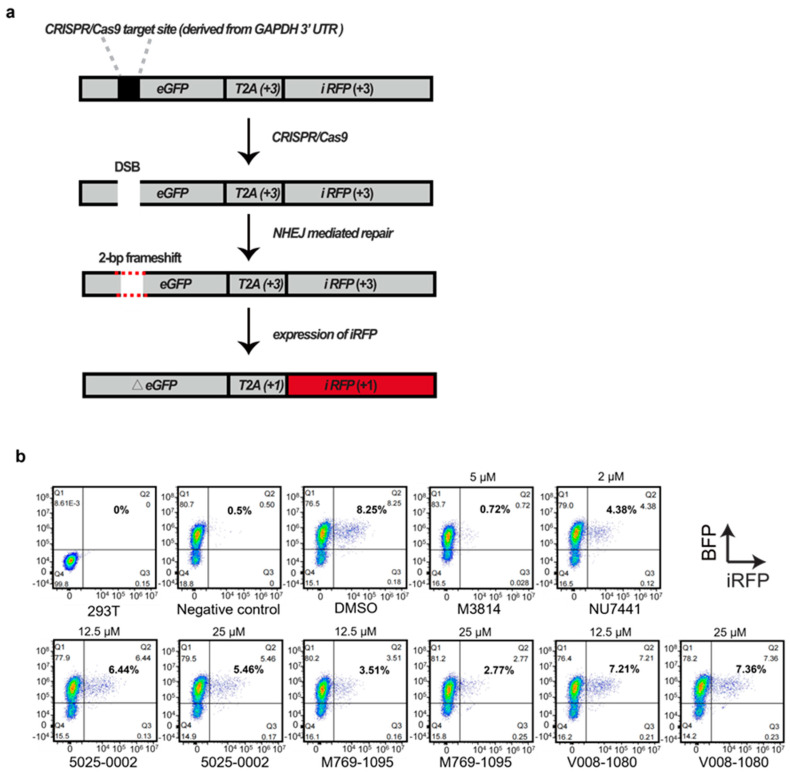
Evaluation of NHEJ efficiency in HEK293T cells treated with different small molecules using modified TLR reporter. (**a**) Schematic depicting the outcome after the induction of a site-specific double-strand break (DSB). If the break undergoes NHEJ mediated repair, eGFP will be translated out of frame and iRFP will be expressed, producing far-red fluorescent cells. (**b**) HEK293T cells overexpressing the TLR reporter (BFP positive) were nucleofected with Cas9 and sgRNA plasmids, then treated with different small molecules or vehicle. NHEJ efficiency was evaluated by detecting iRFP expression 48 h post-treatment using a flow cytometer.

Next, we investigated whether the increase in HDR efficiency by V008-1080 was due to its inhibition of the NHEJ process. The robust ‘Traffic Light Reporter’ (TLR) assay, developed by Scharenberg and colleagues [31], relies on NHEJ-mediated repair to enable mCherry production. We modified the reporter by replacing mCherry with iRFP and the I-Sce1 meganuclease cutting site with the GAPDH 3′-UTR sequence that can be cut by CRISPR/Cas9 system (Figure 6a, Supplementary Figure S5). We tested compound 5025-0002, M769-1095, and V008-1080, along with M3814 and NU7441, for their ability to inhibit NHEJ events by detecting iRFP expression using FACS analysis (Figure 6b).

HEK293T cells expressing the modified TLR reporter, which could also express BFP, were established (Supplementary Figure S5). Cas9/sgRNA expression vectors targeting GAPDH 3′-UTR were nucleofected into the cells. Forty-eight hours later, a flow cytometry analysis of iRFP positive cells revealed that NHEJ events occurred in about 8% of the cells (DMSO group, 8.25%) (Figure 6b). Since only one out of three repair events were expected to yield iRFP positive cells, the actual NHEJ events were about 24%. The nucleofection of TLR-overexpressing HEK293T cells with Cas9/sgRNAs plasmids, followed by M3814 (0.72%) or NU7441 (4.38%) treatment significantly decreased NHEJ events compared to DMSO treatment (8.25%). Notably, the nucleofected cells treated with all three compounds displayed decreased NHEJ events. Given that 5025-0002 and M769-1095 exhibited relatively high cytotoxicity against HEK293T cells (Figure 5b), the more substantial NHEJ event inhibition induced by 5025-0002 (6.44% at 12.5 μM, 5.46% at 25 μM) or M769-1095 (3.51% at 12.5 μM, 2.77% at 25 μM) compared to V008-1080 (7.21% at 12.5 μM, 7.36% at 25 μM) might be due to the high cytotoxicity. As expected, when the concentration of 5025-0002 and M769-1095 were adjusted to 12.5 μM, the NHEJ inhibition effect was significantly relieved (Figure 6b). In contrast, cells treated with V008-1080 at different concentrations showed comparable NHEJ inhibition effects (7.21% at 12.5 μM, 7.36% at 25 μM), indicating that the non-specific cytotoxicity of V008-1080 was weaker than that of the other compounds. Taken together, these results suggest that V008-1080 can increase HDR-mediated knock-in efficiency by inhibiting NHEJ events.

### 2.4. Molecular Docking for Inhibitor and DNA-PKcs

**Figure 7:**
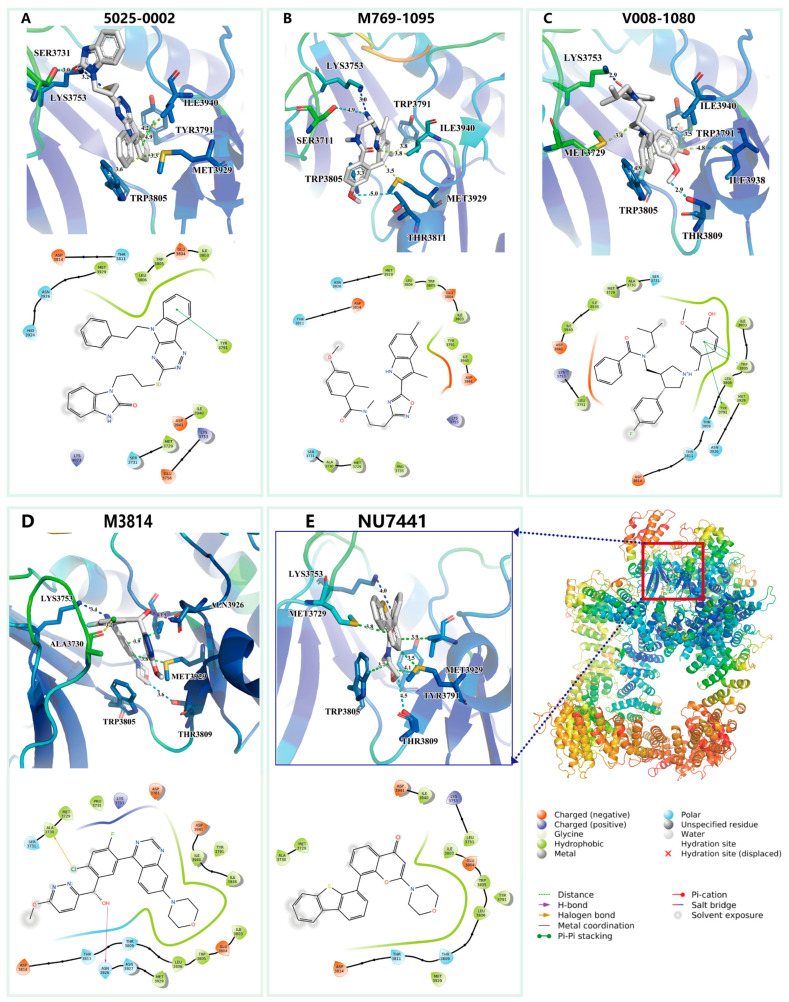
Interaction analysis of DNA-PKcs with newly discovered and known active compounds with docked conformation. (**A**) Compound 5025-0002 binding to the DNA-PKcs pocket, displaying both 3D detailed interactions above and 2D representation below. (**B**) Interaction between M769-1095 and DNA-PKcs pocket. (**C**) Interaction between V008-1080 and DNA-PKcs pocket. (**D**) Interaction between M3814 and DNA-PKcs pocket. (**E**) Interaction between NU7441 and DNA-PKcs pocket. Residues in all 3D visualizations are color-coded by B-Factor in PyMOL, with distances for crucial interactions (depicted with dotted lines) measured. The 2D diagrams employ colors and symbols as standardized by the Schrödinger 2D interaction plots.

We analyzed the detailed interaction between the docked structures of the three identified active compounds and two known active compounds with DNA-PKcs (Figure 7). The primary interactions between 5025-0002 and DNA-PKcs are hydrophobic and electrostatic (Figure 7A). Specifically, the residues MET3929, LEU3806, TRP3805, ILE3803, and TYR3791 predominantly form hydrophobic interactions with the cyclohexane ring of 5025-0002. Additionally, TYR3791 engages in π–π stacking interactions with the benzo-fused five-membered ring of the compound. Similarly, M769-1095 interacts with the DNA-PKcs pocket through hydrophobic, electrostatic, and polar interactions (Figure 7B). The benzo-fused five-membered ring of M769-1095 engages in hydrophobic interactions with the residues TYR3791 and ILE3940. In the interaction between V008-1080 and the DNA-PKcs binding site, multiple pocket residues are implicated (Figure 7C). Notably, ILE3803, TRP3805, LEU3806, and TYR3791 predominantly form hydrophobic contacts with V008-1080. TRP3805 and TYR3791 also establish π–π stacking interactions with the phenol-like ring of V008-1080. Additionally, the negatively charged residue ASP3941 forms a significant π-related interaction with the benzene ring of V008-1080.

For comparison, we also rendered the docking predictions for the binding conformations of two known active compounds, M3814 and NU7441. The interaction between M3814 and the DNA-PKcs pocket is primarily characterized by hydrophobic and electrostatic interactions. Predominantly, TRP3805, LEU3906, and several ILE residues (3940, 3938, 3803) engage in hydrophobic interactions with the six-membered heterocycle and the biphenyl-like structure of M3814. Additionally, ASN3926 forms a hydrogen bond with the hydroxyl group of M3814, and the compound’s chlorine atom engages in a halogen bond with ALA3730. Similarly, NU7441 interacts with the DNA-PKcs pocket primarily through hydrophobic contacts, with its six-membered heterocycle and biphenyl-like structure. It is noteworthy that both M3814 and NU7441 possess a morpholine substructure, which is absent in the three newly identified compounds, suggesting that our methods can recognize diverse molecular architectures.

In summary, all five active compounds extensively engage in hydrophobic interactions with several hydrophobic residues. Moreover, these compounds share several common interaction residues, as indicated by the 2D interaction plots. Residues such as TRP3805, LYS3753, TYR3791, MET3929, ASP3941, GLU3804, and ASP381 are notably involved in important interactions with all five compounds. The presence of these shared interacting residues suggests their critical role in facilitating the successful docking of ligands within the binding pocket.

## 3. Discussion

In the present work, 1,507,824 compounds from the FDA-approved library were explored using deep learning-based algorithms combined with molecular dynamics (MD) simulations. Eight potential DNA-PKcs inhibitors were screened and sent for further experimental validation. Finally, we identified a novel DNA-PKcs inhibitor, V008-1080. It exhibited the specific cytotoxicity against DNA-PKcs overexpressed 786-O human RCC cell line, while showing no cytotoxicity against other cell lines like HK-2 and HEK293T. A subsequent test of the HDR-mediated knock-in test by CRISPR/Cas9 showed that V008-1080 could boost the HDR efficiency at the expense of NHEJ. Compared to M3814 and NU7441, V008-1080 displayed weaker activity. These results indicate that V008-1080 is a weak DNA-PKcs inhibitor and requires structural modifications to enhance its inhibitory activity. We compared the interactions of DNA-PKcs pockets with M3814 and V008-1080. M3814 formed five hydrogen bonds with the DNA-PKcs protein, while M3814 engaged in three π–π interactions and one π–cation interaction. Generally, π–π or π–cation interactions are considered weaker than hydrogen bonds. However, the IC_50_ value of V008-1080 (74.84 μM) is slightly higher than that of M3814 (36.04 μM) (Figure 4j,o). This suggests that the molecular skeleton of V008-1080 may have a preferable conformation for interacting with DNA-PKcs, making it a potential lead compound. The following is a potential modification for compound V008-1080. Firstly, we can replace the fluorine on the B ring with an electron-donating group, such as methoxyl, to facilitate the π–cation interaction of the B ring with ARG3737. Secondly, we can introduce a hydrogen bond receptor on the C ring, such as -Cl, -CN, or carbonyl, or replace the C ring with a pyridine group. Thirdly, we can link -OH and -OCH_3_ on the A ring to form a dioxole group to facilitate the π–π interaction, and introduce -F, -Cl, -CN or ester groups to explore possible hydrogen bond interactions. We believe that these structural optimizations based on compound V008-1080 can facilitate the discovery of more active molecules targeting DNA-PKcs (Supplementary Figure S6). We have proposed a potential modification strategy for V008-1080 and produced 15 derivates based on rings A, B and C (Supplementary Figure S7). We calculated the affinity of the modified compounds by molecular docking, and the scores are listed in the Supplementary Table S1. This analysis indicates the derivates have good binding affinity to DNA-PKcs, except A6 and A3.

DNA-PK is composed of DNA-PKcs and a Ku protein heterodimer (the Ku70 and Ku80 subunits). In our current work, we focused on identifying small molecules targeting DNA-PKcs. Previously, several DNA-PKcs inhibitors have been discovered [32]. These inhibitors can be subdivided into two main categories: ATP-competitive inhibitors and non-competitive inhibitors. ATP-competitive inhibitors compete with ATP for binding to the ATP-binding site of DNA-PKcs and prevent its kinase activity. These inhibitors typically resemble ATP structurally and function by blocking ATP binding and subsequent phosphorylation events. Examples of ATP-competitive inhibitors include NU7441 (also known as KU-57788), M3814 and AZD7648. Non-competitive inhibitors, on the other hand, bind to alternative sites on DNA-PKcs and disrupt its function without directly competing with ATP. These inhibitors may interfere with the interaction between DNA-PKcs and other proteins or block the conformational changes required for DNA repair activity. The ATPγS-binding site of DNA-PKcs includes Glu3804 of the main chain and Leu3751, Tyr3791, TRP3805, LEU3806 and Ile3940 of the side chains, forming a hydrophobic surface [33]. NU7441 and M3814 target the ATP-binding groove of DNA-PKcs, overlapping with the ATPγS-binding site. These small molecules function via direct ATP competition. In our study, 5025-0002, V008-1080 showed significant overlap with ATPγS in the binding groove, including LEU3806, TRP3805 and TYR3791. When a double-strand break (DSB) occurs, DNA-PKcs is responsible for transferring the γ-phosphate from ATP to specific amino acid residues in components of the NHEJ pathway. This phosphorylation facilitates a subsequent DNA repair process. In short, the screened small molecules could competitively bind to ATPγS-binding site, thereby inhibiting the phosphorylation of DNA-PKcs as well as a following DNA repair process via the NHEJ pathway.

Cancer is a significant global health challenge, with increasing incidence and mortality rates worldwide. However, current cancer treatment approaches remain limited, highlighting the urgent need for novel therapies, including discovering new drugs [34,35]. One promising strategy is to discover novel drugs based on identified cancer targets [36,37]. DNA-PK is an attractive tumor target, as its abnormal activity is involved in various types of tumors. DNA-PKcs inhibitors can enhance the efficacy of chemotherapy or radiotherapy [10,12,38]. Previously, Tarazi et al. performed docking-based virtual screening against a database of FDA-approved drugs and identified two potential DNA-PK inhibitors (Praziquantel and Dutasteride), which were validated through biological investigation [39]. The cryo-electron microscopy (cryo-EM) structures of human DNA-PKcs provide detailed molecular insights, facilitating the subsequent virtual screening [33]. Based on this novel structure, we employed our developed hybrid screening strategy to perform high-throughput virtual screening and successfully identified a novel DNA-PKcs inhibitor. This study marks the first instance of deep learning- and molecular simulation-based hybrid screening performed using the accurate structures of human DNA-PKcs. The use of artificial intelligence in drug discovery has gained significant attention in recent years due to its ability to process and analyze vast amounts of data efficiently. By leveraging this technology, we were able to narrow down a large number of compounds to a select few with high predicted binding affinities, saving both time and resources in the drug discovery process.

Our current strategy is to perform virtual screening, targeting the ATP-binding site of DNA-PKcs, as located in the cryo-EM structures. Through this approach, we identified V008-1080 as a weak ATP-competitive inhibitor. Future rounds of virtual screening can focus on the binding sites of non-competitive inhibitors. Since many proteins bind ATP, it is important to determine whether our identified ligand has off-target effects. In future studies, we propose to collect data on various ATP binding proteins and use docking- and deep learning-based methods to assess whether our ligand can bind to other proteins. If our ligand binds to many other proteins, it may have low specificity. Otherwise, its specificity would be high. Additionally, we can explore other sites or the Ku protein site for screening a database of large compounds.

Moreover, potential compounds that likely inhibit the Ku70 or Ku80 subunits could be investigated by virtual screening. Previously, it was found that a new class of DNA-PK inhibitors inhibited the Ku–DNA interaction [40]. Another study identified a small molecule inhibitor that bound directly to the Ku protein heterodimer and inhibited its interaction with DNA, thereby inhibiting the activity of the DNA-PK complex [41]. Therefore, discovering DNA-PK inhibitors with novel mechanisms of action holds promise. Identifying more DNA-PK inhibitors will be beneficial in preclinical studies for their ability to sensitize cancer cells to DNA damage-induced cell death and enhance HDR-mediated gene knock-in efficiency. Currently, six DNA-PK inhibitors are involved in clinical studies due to their ability to enhance the efficacy of chemotherapies or radiotherapies [23]. The discovery of novel DNA-PK inhibitors will contribute to the development of approved anti-tumor drugs in the future.

On the other hand, the cytotoxicity of a candidate should be considered when performing high-throughput virtual screening. In our current study, although we identified several small molecules as potential DNA-PKcs inhibitors through virtual screening, subsequent experimental validation showed some of the compounds exhibited high non-specific cytotoxicity, affecting their potential HDR-mediated gene editing activities. Ultimately, only one compound, V008-1080, with minimal non-specific cytotoxicity, was identified as a potential DNA-PKcs inhibitor. Similarly, in vivo toxicities, including acute toxicity, carcinogenicity, cardiotoxicity, hepatotoxicity, and mutagenicity should be evaluated before a compound is advanced to clinical study. In the future, a deep learning approach can be employed to identify or exclude potential toxic candidates, including but not limited to small molecules.

In conclusion, our study successfully employed deep learning-based screening, experimental validation, and molecular dynamics simulations to identify active compounds against DNA-PKcs. The results highlight the potential of these compounds as promising anticancer therapeutics. Nonetheless, further research is necessary to optimize and validate these compounds and explore their broader implications in cancer therapy.

## 4. Conclusions

In conclusion, our screening and experimental validation of active compounds against DNA-PKcs have yielded promising results and provided valuable insights into the development of potential anticancer therapeutics. Utilizing our deep learning-based drug screening pipeline, we efficiently narrowed down a large pool of compounds to a select few that exhibited high predicted binding affinities against DNA-PKcs. The integration of deep learning algorithms has proven to be a valuable tool in prioritizing and identifying potential drug candidates, enabling a more efficient and targeted approach in the drug discovery process. To validate the predicted binding affinities, we performed experimental studies to assess the binding interactions and biological activity of the selected compounds against DNA-PKcs. The results demonstrated that one of the compounds showed significant inhibition of DNA-PKcs activity, validating the accuracy of the predictions made by our screening pipeline.

Additionally, our study involved applying molecular dynamics (MD) simulations to gain insights into the dynamic behavior and stability of the protein–ligand complexes. The MD simulations provided valuable information regarding the binding mechanisms, including the formation of key interactions and conformational changes induced upon binding. This dynamic information further supported the selection and prioritization of the most promising drug candidates. The successful integration of deep learning-based screening, experimental validation, and MD simulations has accelerated the drug discovery process, enabling the identification of active compounds against DNA-PKcs. These compounds have demonstrated potential as anticancer therapeutics, with their ability to inhibit DNA-PKcs activity, a key enzyme involved in DNA repair and essential for cancer cell survival. Our findings lay the foundation for the further optimization and development of these active compounds as potential drugs. The knowledge gained from this study contributes to the understanding of DNA-PKcs inhibition and provides insights into targeting DNA repair pathways in cancer therapy.

In summary, our screening and experimental validation of active compounds against DNA-PKcs have successfully identified a potential anticancer effect and HDR promotion activity. The integration of deep learning-based screening, experimental validation, and MD simulations has facilitated a comprehensive understanding of the binding mechanisms and the stability of protein–ligand complexes. This integrated approach holds great promise for the accelerated discovery of effective treatments against DNA-PKcs and potentially other molecular targets relevant to cancer and other diseases.

## 5. Materials and Methods

### 5.1. Protein Acquisition and Pocket Identification

The screening process began with a deep learning approach combined with molecular dynamics (MD) simulations. The atomic coordinates of DNA-PKcs were retrieved from PDB with ID 7OTY [33]. The PDB structure already contained the known ligand Nedisertib (PDB ligand ID 1IX). The pocket was defined as the region within 1 nm of the predicted or known ligand. The Chemdiv dataset, comprising 1,507,824 compounds, was used as a virtual screening dataset.

### 5.2. Screening by DeepBindGCN

The DeepBindGCN_BC and DeepBindGCN_RG models, developed in our previous work [26], are designed for fast lead virtual screening tasks. DeepBindGCN_BC is a binary classification model, with prediction values ranging from 0 to 1. An output value closer to 1 indicates a high probability of the protein–ligand pair binding, efficiently distinguishing between binding and nonbinding pairs. We used a cutoff distance of 0.6 nm for DeepBindGCN_BC, as in our previous study, where any residue within this distance of the ligand was considered part of the pocket residue. However, DeepBindGCN_BC cannot distinguish between weak binders and strong affinity binders. DeepBindGCN_RG was designed to predict the affinity of protein–ligand binding with real value as the output, making it suitable for distinguishing weak and strong binders. The protein pocket was defined as within 0.8 nm of the known ligand for DeepBindGCN_RG. In both DeepBindGCN_BC and DeepBindGCN_RG, ligands were represented as molecular graphs by converting their SMILES code to the corresponding molecular graph and extracting atomic features using the open-source chemical informatics software RDKit, version 2023.3.2 [42]. The protein pocket was represented as a graph by considering residues as nodes and identifying contacting residue pairs as edges, with a cutoff distance of 0.5 nm for defining contacts.

To generate the molecular vectors of chemical groups, a pre-trained mol2vec model was employed, yielding a dimension of 30 [43]. Standard amino acid vectors were represented by adding their respective chimerical group vectors. For small molecules, atoms were encoded using a one-hot-like representation similar to the GraphDTA method [44]. Notably, DeepBindGCN_RG predicts the pKa value, transforming binding affinities into pKa using the following equation:

pKa = −log_10_ K_x_

where K_x_ represents IC_50_, K_i_, or K_d_.

In this work, we used DeepBindGCN_BC and DeepBindGCN_RG to screen the Chemdiv database. Candidates for late-stage docking were selected based on DeepBindGCN_BC scores of ≥0.99 and DeepBindGCN_RG scores of ≥9.0.

### 5.3. Schrödinger Docking Process

First, the ligands underwent geometric optimization using the Ligprep module. The ligand energy was minimized using the OPLS 2005 force field, and all its ionized states were generated at pH 7.4. Subsequently, a single, low-energy three-dimensional structure of the ligand was generated while preserving its original chiral state. Hydrogen atoms were added to the protein, and the system was optimized at pH 7.4 using the OPLS-u-2005 force field. The receptor grid was generated based on the geometric center coordinates of the protein’s ligands. We used van der Waals radius scaling, with a scaling factor of 1 and a partial charge cutoff of 0.25. The centroid of the workspace ligand was chosen as the center. The docked ligand was confined to the enclosing box. The enclosing box was defined as residues within a cutoff of 1.6 nm from the 1IX ligand in PDB structure (PDB ID: 7OTY),and a final grid box with dimensions of 26 Å × 26 Å × 26 Å was obtained. No constraints, rotatable groups or excluded volumes were used.

### 5.4. Force Field-Based Screening

In the drug screening process, additional screening was performed using force field-based molecular dynamics (MD) simulations. Specifically, 19 compound binding complexes, identified as predicted candidates by previous deep learning and docking methods, and the criteria of DeepBindGCN_BC score ≥ 0.99, DeepBindGCN_RG score ≥ 9, and Schrödinger docking energy ≤ −7 kcal/mol were selected for MD simulations. The goal of these simulations was to calculate the binding free energy for each complex. Metadynamics simulations were employed to estimate the binding free energy by sampling the free energy landscape of the protein–ligand complexes in solution. Metadynamics involves the addition of a bias potential to facilitate the exploration of the free energy landscape along a specific collective variable of interest. By employing this technique, the likelihood of protein–ligand binding in solution can be further evaluated [45]. The detailed MD simulation, along with the subsequent metadynamics simulation, is extensively described in Supplementary Materials, Section S1.

### 5.5. Cell Culture

As previously reported [29], human RCC cell lines (786-O) and normal human adult male kidney cells (HK-2) were obtained from Shanghai Biological Institute (Shanghai, China). HEK293T cells were obtained from ATCC. Both cells were cultured in Dulbecco’s modified Eagle’s medium (DMEM), supplemented with 10% fetal bovine serum (FBS), 100 U/mL penicillin, and 100 μg/mL streptomycin. The cells were dissociated using 0.25% EDTA-trypsin and passaged every 2–3 days at a ratio of 1:3-5.

### 5.6. Nucleofection

HEK293T cells were harvested with 0.25% trypsin (Life Technologies, Carlsbad, California, USA), and centrifuged at 300× *g* for 5 min at room temperature. Then, 1 × 10^6^ cells were resuspended in 100 µL primary cell nucleofection solution (P2 Primary Cell 4D-Nucleofector X kit S) [12 RCT, V4XP-2032; Lonza]. A total of 5 µg Cas9 protein (TrueCut™ Cas9 Protein v2, Thermo Fisher, Waltham, MA, USA) and 10 µg sgRNA (generated by GeneArt™ Precision gRNA Synthesis Kit, Thermo Fisher, Waltham, MA, USA) were mixed and incubated for 10 min before nucleofection with the cells and DNA donors in the Nucleofector 4D Device. Alternatively, Cas9 plasmid and sgRNA plasmid were nucleofected with the cells. The cells were then resuspended in DMEM and aliquoted into a 48-well plate at 400 μL per well. DMSO and different small molecules were then added to the nucleofected cells.

### 5.7. Evaluation of Knock-In Events

HEK293T cells were co-nucleofected with the ires-GFP donor and RNP system using a Nucleofector 4D Device. Then, the nucleofected cells were treated with small molecules at the indicated concentrations for 48 h. After treatment, the cells were harvested with 0.25% trypsin, washed with PBS, and then stained with propidium iodide (PI), using an apoptosis detection kit for FACS analysis. The expression of GFP protein allowed for the direct assessment of HDR-mediated knock-in efficiency. The gates for negative cells were set based upon values of untransfected HEK293T cells. All samples were analyzed using a flow cytometer (CytoFLEX, BECKMAN COULTER, Brea, CA, USA).

### 5.8. Traffic Light Reporter (TLR) Assay

The TLR assay was performed essentially as described [31]. The TLR plasmid (Addgene #31481) was modified by replacing mCherry with iRFP, and the I-Sce1 meganuclease cutting site with the GAPDH 3′-UTR. The presence of blue fluorescent protein (BFP) in the modified TLR plasmid allowed for corrections for transfection efficiencies. The modified TLR plasmid was packaged into lentivirus to infect HEK293T cells. Then, 1–2 μg Cas9 plasmid (Addgene # 41815) and 0.5–1 μg sgRNA, targeting GAPDH 3′-UTR site, were nucleofected into the TLR-infected HEK293T cells with a Nucleofector 4D Device. NHEJ efficiency was evaluated by detecting iRFP expression using a flow cytometer. The values obtained by quantitating the iRFP+ cells were multiplied by three since only one out of three repair events is expected to yield a ΔeGFP-T2A-iRFP fusion in the correct frame to generate iRFP+ cells.

### 5.9. Cell Proliferation Assay

786-O RCC cells were seeded into 96-well clear, flat-bottom plates at a density of 5000 cells per well. The cells were then incubated at 37 °C with 5% CO_2_. Following an overnight incubation, a serial dilution of compounds or 1% DMSO was prepared and added to the cells. After 72 h of incubation, the cells were washed with 1×PBS and subsequently incubated with fresh medium, supplemented with CCK8 (TOPSCIENCE, Shanghai, China) for 1 h according to the manufacturer’s instructions. To assess cell proliferation, we measured the light absorption value at 450 nm. Each experiment was conducted in triplicate, and the data were analyzed using GraphPad Prism software, version 10.2.3. Cell viability (%) was calculated as follows: Cell viability (%) = 100% × (absorbance of treated cells − absorbance of background controls)/(absorbance of DMSO controls − absorbance of background controls). To determine cell inhibition (%), we employed the formula: Cell inhibition (%) = 1 − Cell viability (%). Finally, the IC_50_ values were calculated through nonlinear regression analysis.

### 5.10. Statistical Analysis

Statistical analysis was performed using GraphPad Prism software (version 8.0.2, GraphPad Software, USA). The mean and standard deviation (SD) were calculated for the data. Statistical differences between experimental groups were analyzed using Student’s *t*-test. All data are presented as mean ± SD, with statistical significance defined as *p* < 0.05.

## Figures and Tables

**Table 1:** Candidate compounds from deep learning and docking screening. We only selected those candidates with DeepBindGCN_BC ≥ 0.99, DeepBindGCN_RG ≥ 9, and Schrödinger docking energy ≤ −7 Kcal/mol.

Chemdiv ID	DeepBindGCN_BC	DeepBindGCN_RG	Schrödinger Docking (Kcal/mol)
L102-0385	0.9988	9.1991	−8.4419
8601-0106	1.0000	9.1074	−8.0588
5795-0108	1.0000	9.3339	−7.9719
C684-0025	0.9985	9.0321	−7.9543
4290-0112	0.9959	9.0631	−7.8938
V008-1080	0.9983	9.3616	−7.7966
C200-6885	0.9975	9.0352	−7.5277
C163-0038	1.0000	9.4493	−7.4668
V014-8131	1.0000	9.1249	−7.4660
E208-0020	1.0000	9.1201	−7.4659
S431-0991	0.9999	9.1192	−7.4172
C163-0087	1.0000	9.0191	−7.3098
V001-2119	0.9947	9.0161	−7.2953
G744-0225	0.9996	9.0588	−7.2909
SA50-0140	1.0000	9.1702	−7.2194
C163-0039	1.0000	9.4007	−7.1913
5025-0002	0.9999	9.0076	−7.1717
7238-1541	0.9986	9.1096	−7.1709
M769-1095	0.9936	9.0299	−7.1306

## Data Availability

The proposed models and scripts are available upon request from the corresponding author.

## Supplementary Materials

The following supporting information can be downloaded at: https://www.mdpi.com/article/10.3390/ijms25147982/s1. References [46,47,48,49,50,51,52,53,54,55,56,57,58] are cited in the Supplementary Materials.

## References

[1] Carter T., Vancurova I., Sun I., Lou W., DeLeon S. (1990). A DNA-activated protein kinase from hela cell nuclei. Mol. Cell Biol..

[2] Jackson S.P., MacDonald J.J., Lees-Miller S., Tjian R. (1990). Gc box binding induces phosphorylation of sp1 by a DNA-dependent protein kinase. Cell.

[3] Lees-Miller S.P., Chen Y.R., Anderson C.W. (1990). Human cells contain a DNA-activated protein kinase that phosphorylates simian virus 40 t antigen, mouse p53, and the human ku autoantigen. Mol. Cell Biol..

[4] Aparicio T., Baer R., Gautier J. (2014). DNA double-strand break repair pathway choice and cancer. DNA Repair.

[5] Ceccaldi R., Rondinelli B., D’Andrea A.D. (2016). Repair pathway choices and consequences at the double-strand break. Trends Cell Biol..

[6] Yue X., Bai C., Xie D., Ma T., Zhou P.K. (2020). DNA-pkcs: A multi-faceted player in DNA damage response. Front. Genet..

[7] Jackson S.P. (1997). DNA-dependent protein kinase. Int. J. Biochem. Cell Biol..

[8] Goodwin J.F., Knudsen K.E. (2014). Beyond DNA repair: DNA-pk function in cancer. Cancer Discov..

[9] Ciszewski W.M., Tavecchio M., Dastych J., Curtin N.J. (2014). DNA-pk inhibition by nu7441 sensitizes breast cancer cells to ionizing radiation and doxorubicin. Breast Cancer Res. Treat..

[10] Fok J.H.L., Ramos-Montoya A., Vazquez-Chantada M., Wijnhoven P.W.G., Follia V., James N., Farrington P.M., Karmokar A., Willis S.E., Cairns J. (2019). Azd7648 is a potent and selective DNA-pk inhibitor that enhances radiation, chemotherapy and olaparib activity. Nat. Commun..

[11] Zenke F.T., Zimmermann A., Sirrenberg C., Dahmen H., Kirkin V., Pehl U., Grombacher T., Wilm C., Fuchss T., Amendt C. (2020). Pharmacologic inhibitor of DNA-pk, m3814, potentiates radiotherapy and regresses human tumors in mouse models. Mol. Cancer Ther..

[12] Smithson M., Irwin R.K., Williams G., McLeod M.C., Choi E.K., Ganguly A., Pepple A., Cho C.S., Willey C.D., Leopold J. (2022). Inhibition of DNA-pk may improve response to neoadjuvant chemoradiotherapy in rectal cancer. Neoplasia.

[13] Bibikova M., Carroll D., Segal D.J., Trautman J.K., Smith J., Kim Y.G., Chandrasegaran S. (2001). Stimulation of homologous recombination through targeted cleavage by chimeric nucleases. Mol. Cell Biol..

[14] Choulika A., Perrin A., Dujon B., Nicolas J.F. (1995). Induction of homologous recombination in mammalian chromosomes by using the i-scei system of saccharomyces cerevisiae. Mol. Cell Biol..

[15] Sander J.D., Cade L., Khayter C., Reyon D., Peterson R.T., Joung J.K., Yeh J.R. (2011). Targeted gene disruption in somatic zebrafish cells using engineered talens. Nat. Biotechnol..

[16] Cong L., Ran F.A., Cox D., Lin S., Barretto R., Habib N., Hsu P.D., Wu X., Jiang W., Marraffini L.A. (2013). Multiplex genome engineering using crispr/cas systems. Science.

[17] Mali P., Yang L., Esvelt K.M., Aach J., Guell M., DiCarlo J.E., Norville J.E., Church G.M. (2013). Rna-guided human genome engineering via cas9. Science.

[18] Robert F., Barbeau M., Ethier S., Dostie J., Pelletier J. (2015). Pharmacological inhibition of DNA-pk stimulates cas9-mediated genome editing. Genome Med..

[19] Wimberger S., Akrap N., Firth M., Brengdahl J., Engberg S., Schwinn M.K., Slater M.R., Lundin A., Hsieh P.P., Li S. (2023). Simultaneous inhibition of DNA-pk and polϴ improves integration efficiency and precision of genome editing. Nat. Commun..

[20] Riesenberg S., Chintalapati M., Macak D., Kanis P., Maricic T., Paabo S. (2019). Simultaneous precise editing of multiple genes in human cells. Nucleic Acids Res..

[21] Jekimovs C., Bolderson E., Suraweera A., Adams M., O’Byrne K.J., Richard D.J. (2014). Chemotherapeutic compounds targeting the DNA double-strand break repair pathways: The good, the bad, and the promising. Front. Oncol..

[22] Gavande N.S., VanderVere-Carozza P.S., Hinshaw H.D., Jalal S.I., Sears C.R., Pawelczak K.S., Turchi J.J. (2016). DNA repair targeted therapy: The past or future of cancer treatment?. Pharmacol. Ther..

[23] Hu S., Hui Z., Lirussi F., Garrido C., Ye X.Y., Xie T. (2021). Small molecule DNA-pk inhibitors as potential cancer therapy: A patent review (2010-present). Expert. Opin. Ther. Pat..

[24] Zhang H., Saravanan K.M., Zhang J.Z.H. (2023). Deepbindgcn: Integrating molecular vector representation with graph convolutional neural networks for protein-ligand interaction prediction. Molecules.

[25] Zhang H., Yang Y., Li J., Wang M., Saravanan K.M., Wei J., Tze-Yang Ng J., Tofazzal Hossain M., Liu M., Zhang H. (2020). A novel virtual screening procedure identifies pralatrexate as inhibitor of SARS-CoV-2 rdrp and it reduces viral replication in vitro. PLoS Comput. Biol..

[26] Zhang H., Fan H., Wang J., Hou T., Saravanan K.M., Xia W., Kan H.W., Li J., Zhang J.Z.H., Liang X. (2024). Revolutionizing gpcr-ligand predictions: Deepgpcr with experimental validation for high-precision drug discovery. Brief. Bioinform..

[27] Chen Y., Zhang L., Graf L., Yu B., Liu Y., Kochs G., Zhao Y., Gao S. (2017). Conformational dynamics of dynamin-like mxa revealed by single-molecule fret. Nat. Commun..

[28] Hu J.L., Liang H., Zhang H., Yang M.Z., Sun W., Zhang P., Luo L., Feng J.X., Bai H., Liu F. (2020). Fam46b is a prokaryotic-like cytoplasmic poly(a) polymerase essential in human embryonic stem cells. Nucleic Acids Res..

[29] Zheng B., Mao J.H., Li X.Q., Qian L., Zhu H., Gu D.H., Pan X.D. (2016). Over-expression of DNA-pkcs in renal cell carcinoma regulates mtorc2 activation, hif-2alpha expression and cell proliferation. Sci. Rep..

[30] He X., Tan C., Wang F., Wang Y., Zhou R., Cui D., You W., Zhao H., Ren J., Feng B. (2016). Knock-in of large reporter genes in human cells via crispr/cas9-induced homology-dependent and independent DNA repair. Nucleic Acids Res..

[31] Certo M.T., Ryu B.Y., Annis J.E., Garibov M., Jarjour J., Rawlings D.J., Scharenberg A.M. (2011). Tracking genome engineering outcome at individual DNA breakpoints. Nat. Methods.

[32] Leahy J.J., Golding B.T., Griffin R.J., Hardcastle I.R., Richardson C., Rigoreau L., Smith G.C. (2004). Identification of a highly potent and selective DNA-dependent protein kinase (DNA-pk) inhibitor (nu7441) by screening of chromenone libraries. Bioorg Med. Chem. Lett..

[33] Liang S., Thomas S.E., Chaplin A.K., Hardwick S.W., Chirgadze D.Y., Blundell T.L. (2022). Structural insights into inhibitor regulation of the DNA repair protein DNA-pkcs. Nature.

[34] Zhang H., Zhang S.H., Hu J.L., Wu Y.T., Ma X.Y., Chen Y., Yu B., Liao S., Huang H., Gao S. (2021). Structural and functional characterization of multiple myeloma associated cytoplasmic poly(a) polymerase fam46c. Cancer Commun..

[35] He Z., Tian T., Guo D., Wu H., Chen Y., Zhang Y., Wan Q., Zhao H., Wang C., Shen H. (2014). Cytoplasmic retention of a nucleocytoplasmic protein tbc1d3 by microtubule network is required for enhanced egfr signaling. PLoS ONE.

[36] Chen D.Q., Xie Y., Cao L.Q., Fleishman J.S., Chen Y., Wu T., Yang D.H. (2024). The role of abcc10/mrp7 in anti-cancer drug resistance and beyond. Drug Resist. Updat..

[37] Liu J., Fan H., Liang X., Chen Y. (2023). Polycomb repressor complex: Its function in human cancer and therapeutic target strategy. Biomed. Pharmacother..

[38] Moyret-Lalle C., Prodhomme M.K., Burlet D., Kashiwagi A., Petrilli V., Puisieux A., Seimiya H., Tissier A. (2022). Role of emt in the DNA damage response, double-strand break repair pathway choice and its implications in cancer treatment. Cancer Sci..

[39] Tarazi H., Saleh E., El-Awady R. (2016). In-silico screening for DNA-dependent protein kinase (DNA-pk) inhibitors: Combined homology modeling, docking, molecular dynamic study followed by biological investigation. Biomed. Pharmacother..

[40] Gavande N.S., VanderVere-Carozza P.S., Pawelczak K.S., Mendoza-Munoz P., Vernon T.L., Hanakahi L.A., Summerlin M., Dynlacht J.R., Farmer A.H., Sears C.R. (2020). Discovery and development of novel DNA-pk inhibitors by targeting the unique ku-DNA interaction. Nucleic Acids Res..

[41] Pawelczak K.S., Gavande N.S., VanderVere-Carozza P.S., Turchi J.J. (2018). Modulating DNA repair pathways to improve precision genome engineering. ACS Chem. Biol..

[42] Rdkit: Open-Source Cheminformatics Software. https://rdkit.org/.

[43] Jaeger S., Fulle S., Turk S. (2018). Mol2vec: Unsupervised machine learning approach with chemical intuition. J. Chem. Inf. Model..

[44] Nguyen T., Le H., Quinn T.P., Nguyen T., Le T.D., Venkatesh S. (2021). Graphdta: Predicting drug-target binding affinity with graph neural networks. Bioinformatics.

[45] Laio A., Gervasio F.L. (2008). Metadynamics: A method to simulate rare events and reconstruct the free energy in biophysics, chemistry and material science. Rep. Prog. Phys..

[46] Schrödinger L., DeLano W. (2020). PyMOL. http://www.pymol.org/pymol.

[47] Saleh N., Ibrahim P., Saladino G., Gervasio F.L., Clark T. (2017). An Efficient Metadynamics-Based Protocol To Model the Binding Affinity and the Transition State Ensemble of G-Protein-Coupled Receptor Ligands. J. Chem. Inf. Model..

[48] Ruiz-Carmona S., Schmidtke P., Luque F.J., Baker L., Matassova N., Davis B., Roughley S., Murray J., Hubbard R., Barril X. (2017). Dynamic undocking and the quasi-bound state as tools for drug discovery. Nat. Chem..

[49] Hess B., Kutzner C., Van Der Spoel D. (2008). GROMACS 4: Algorithms for highly efficient, load-balanced, and scalable molecular simulation. J. Chem. Theory Comput..

[50] Hornak V., Simmerling C. (2003). Generation of accurate protein loop conformations through low-barrier molecular dynamics. Proteins Struct. Funct. Genet..

[51] Sousa Da Silva A.W., Vranken W.F. (2012). ACPYPE—AnteChamber PYthon Parser interfacE. BMC Res. Notes.

[52] Wang J., Wang W., Kollman P.A., Case D.A. (2006). Automatic atom type and bond type perception in molecular mechanical calculations. J. Mol. Graph. Model..

[53] Jorgensen W.L., Chandrasekhar J., Madura J.D., Impey R.W., Klein M.L. (1983). Comparison of simple potential functions for simulating liquid water. J. Chem. Phys..

[54] Van Der Spoel D., Lindahl E., Hess B., Groenhof G., Mark A.E., Berendsen H.J.C. (2005). GROMACS: Fast, flexible, and free. J. Comput. Chem..

[55] Darden T., York D., Pedersen L. (1993). Particle mesh Ewald: An N log( N ) method for Ewald sums in large systems. J. Chem. Phys..

[56] Hess B., Bekker H., Berendsen H.J.C., Fraaije J.G.E.M. (1997). LINCS: A linear constraint solver for molecular simulations. J. Comput. Chem..

[57] Tribello G.A., Bonomi M., Branduardi D., Camilloni C., Bussi G. (2014). PLUMED 2: New feathers for an old bird. Comput. Phys. Commun..

[58] Williams T., Kelley C., Bröker H.-B., Campbell J., Cunningham R., Denholm D., Elber G., Fearick R., Grammes C., Hart L. (2012). gnuplot 4.6. An Interactive Plotting Program. http://gnuplot.sourceforge.net/.

