# Supplementary material for: Identification and Validation of New DNA-PKcs Inhibitors through High-Throughput Virtual Screening and Experimental Verification
Source: Int J Mol Sci. 2024 Jul 22;25(14):7982. doi: 10.3390/ijms25147982 (PMC11277333; doi:10.3390/ijms25147982)
Supplement: Supplementary file 1 [file ijms-25-07982-s001.zip › ijms-3072134-supplementary.docx]

**Supplementary Information**

Identification and Validation of New DNA-PKcs Inhibitors through High-Throughput Virtual Screening and Experimental Verification

Liujiang Dai^1,2#^, Pengfei Yu^3#^, Hongjie Fan^3^, Wei Xia^4^, Yaopeng Zhao^3,5^, Pengfei Zhang^6^, John Z.H. Zhang^4^, Haiping Zhang^4^* and Yang Chen^3,5^*

**Supplementary material section S1**

**Detailed procedure of pocket MD and metadynamics simulation**

The initial protein-compound complexes were from the top score conformation Schrödinger docking. The ligand was edited by pymol software [1] to make it in the correct protonation state at pH 7.

To save the computational resources, we have carried pocket MD for DNA-PKcs-compound complexes by only keeping the binding pocket region for simulation. Metadynamics simulations can estimate binding free energy calculation to explore whether protein-ligand will bind in solution. Metadynamics relies on adding a bias potential to sample the free energy landscape along a specific collective variable of interest [2],[3]. Note that the binding free energy calculations from Metadynamics may only be suitable for detecting the general trend of binding in virtual screening.

The pocket MD is the same as the classical MD simulation, except that we only use the pocket region to reduce system size for simulation [4], which is inspired by a previous dynamic undocking (DUck) method [5]. An in-house script was used to extract the pocket region of the protein (here, we used 0.8nm within the binding ligand), and the N terminal and C terminal ends were capped with the ACE and NHE terminals, respectively. We applied position restraints to the ACE and NHE terminals to maintain the relative conformation of the pocket. pocket MD simulation was carried out by Gromacs with AMBER-99 force field [6,7]. The topology of the ligand and the partial charges of the ligand were generated by ACPYPE [8], which relies on Antechamber [9]. Firstly, we created a dodecahedron box and put the target-ligand complex at the center. A minimum distance from the protein to the box edge was set to 1 nm. We filled the dodecahedron box with TIP3P water molecules [10], and the counter ions were added to neutralize the total charge using the Gromacs program tool [11]. The long-range electrostatic interactions under the periodic boundary conditions were calculated with the Particle Mesh Ewald approach [12]. A cutoff of 10 Å was used for van der Waals non-bonded interactions. Covalent bonds involving hydrogen atoms were constrained by applying the LINCS algorithm [13].

We performed the energy minimization steps with a step-size of 0.001ns, 100 ps simulation with an isothermal-isovolumetric ensemble (NVT), and 10ns simulation with the isothermal-isobaric ensemble (NPT) for water equilibrium. After that, a 40ns NPT production run (step size 2 fs) was carried out. The Parrinello-Rahman barostat and the modified Berendsen thermostat were used for simulation with a fixed temperature of 308 K and a pressure of 1 atm. RMSD and hydrogen bond number of the trajectory were calculated using Gromacs tools.

The simulation continued using the metadynamics approach to explore the free energy landscape. We carried 40ns metadynamics simulation with Plumed[14] patched Gromacs. The protein-ligand complex's interface coordination number of atoms was used as a collective variable (CV). The protein-ligand interface coordination numbers correlate with the numbers of atom contact, and a larger coordination number usually indicates that the protein-ligand is binding.

The coordination number C is defined as follows by Plumed:

$C=\sum_{i\in A}\sum_{j\in B}S_{ij}$ (S1) and

$S_{ij}=\frac{1-\left(\frac{r_{ij}-d_{0}}{r_{0}}\right)^{n}}{1-\left(\frac{r_{ij}-d_{0}}{r_{0}}\right)^{m}}$ (S2)

In the simulation, n was 8, m was 12, $d_{0}$ was 0 nm, and $r_{0}$ was 0.25 nm. $d_{0}$ is a parameter of the switching function. $r_{ij}$ is the distance between atom i and atom j. The degrees of contact between two groups of atoms can be estimated by the above function(1) [14]. Metadynamics simulation for each protein-ligand system was performed for 40 ns. During the metadynamics simulation, Gaussian values were deposited every 1 ps with a height of 0.3 kJ/mol. The widths of the Gaussians were 5 for the coordination number. The free energy landscapes of the metadynamics simulations along the CV were generated by the Plumed program and plotted using Gnuplot [15].

**Reference:**

1. DeLano WL. Pymol: An open-source molecular graphics tool. CCP4 Newsletter On Protein Crystallography 2002;

2. Laio A, Gervasio FL. Metadynamics: a method to simulate rare events and reconstruct the free energy in biophysics, chemistry and material science. Reports on Progress in Physics 2008; 71:126601

3. Saleh N, Ibrahim P, Saladino G, et al. An Efficient Metadynamics-Based Protocol To Model the Binding Affinity and the Transition State Ensemble of G-Protein-Coupled Receptor Ligands. Journal of Chemical Information and Modeling 2017; 57:1210–1217

4. Zhang H, Yang Y, Li J, et al. A novel virtual screening procedure identifies Pralatrexate as inhibitor of SARS-CoV-2 RdRp and it reduces viral replication in vitro. PLoS computational biology 2020; 16:e1008489

5. Ruiz-Carmona S, Schmidtke P, Luque FJ, et al. Dynamic undocking and the quasi-bound state as tools for drug discovery. Nature Chemistry 2017;

6. Hess B, Kutzner C, Spoel D Van Der. GROMACS 4: algorithms for highly efficient, load-balanced, and scalable molecular simulation. Journal of chemical 2008;

7. Hornak V, Simmerling C. Generation of accurate protein loop conformations through low-barrier molecular dynamics. Proteins: Structure, Function and Genetics 2003;

8. Sousa Da Silva AW, Vranken WF. ACPYPE - AnteChamber PYthon Parser interfacE. BMC Research Notes 2012;

9. Wang J, Wang W, Kollman PA, et al. Automatic atom type and bond type perception in molecular mechanical calculations. Journal of Molecular Graphics and Modelling 2006; 25:247–260

10. Jorgensen WL, Chandrasekhar J, Madura JD, et al. Comparison of simple potential functions for simulating liquid water. The Journal of Chemical Physics 1983; 79:926–935

11. Van Der Spoel D, Lindahl E, Hess B, et al. GROMACS: Fast, flexible, and free. Journal of Computational Chemistry 2005;

12. Darden T, York D, Pedersen L. Particle mesh Ewald: An N ⋅log( N ) method for Ewald sums in large systems. The Journal of Chemical Physics 1993; 98:10089–10092

13. Hess B, Bekker H, Berendsen HJC, et al. LINCS: A linear constraint solver for molecular simulations. Journal of Computational Chemistry 1997; 18:1463–1472

14. Tribello GA, Bonomi M, Branduardi D, et al. PLUMED 2: New feathers for an old bird. Computer Physics Communications 2014;

15. Williams T, Kelley C, Campbell J, et al. Gnuplot 4.6. Software Manual 2012;

**Supplementary Figures**

**
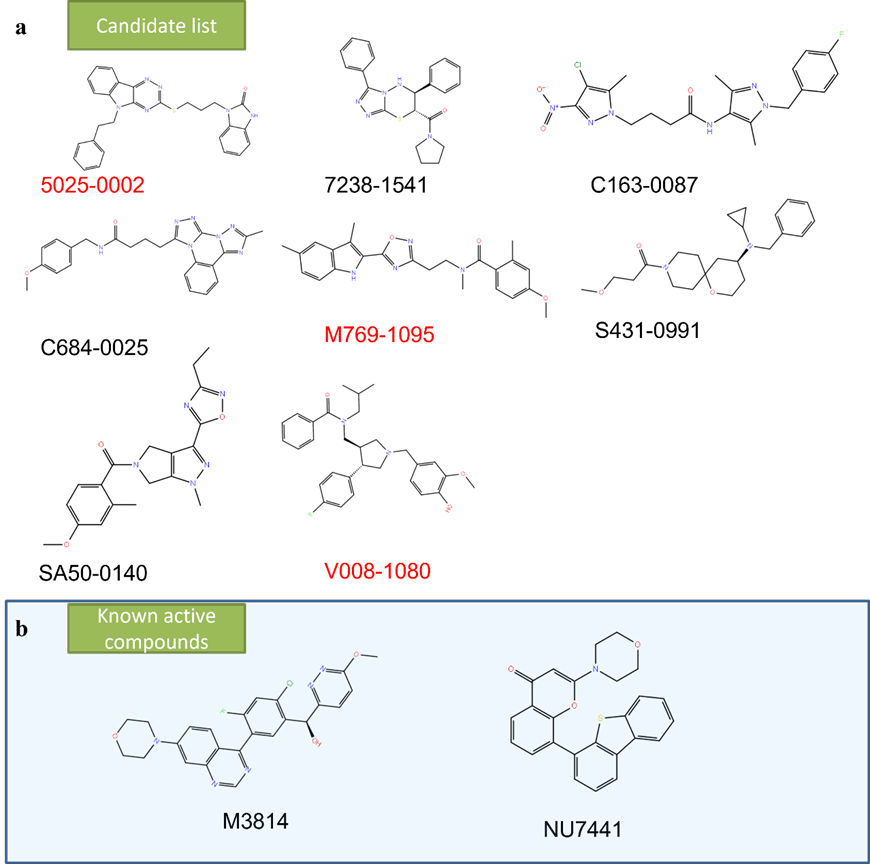
**

**Supplementary Figure S1. 2D structures of different small molecules. (a)** 2D structures of screened eight candidates which were potential DNA-PKcs inhibitors. **(b)** 2D structures of known DNA-PKcs inhibitors.

**
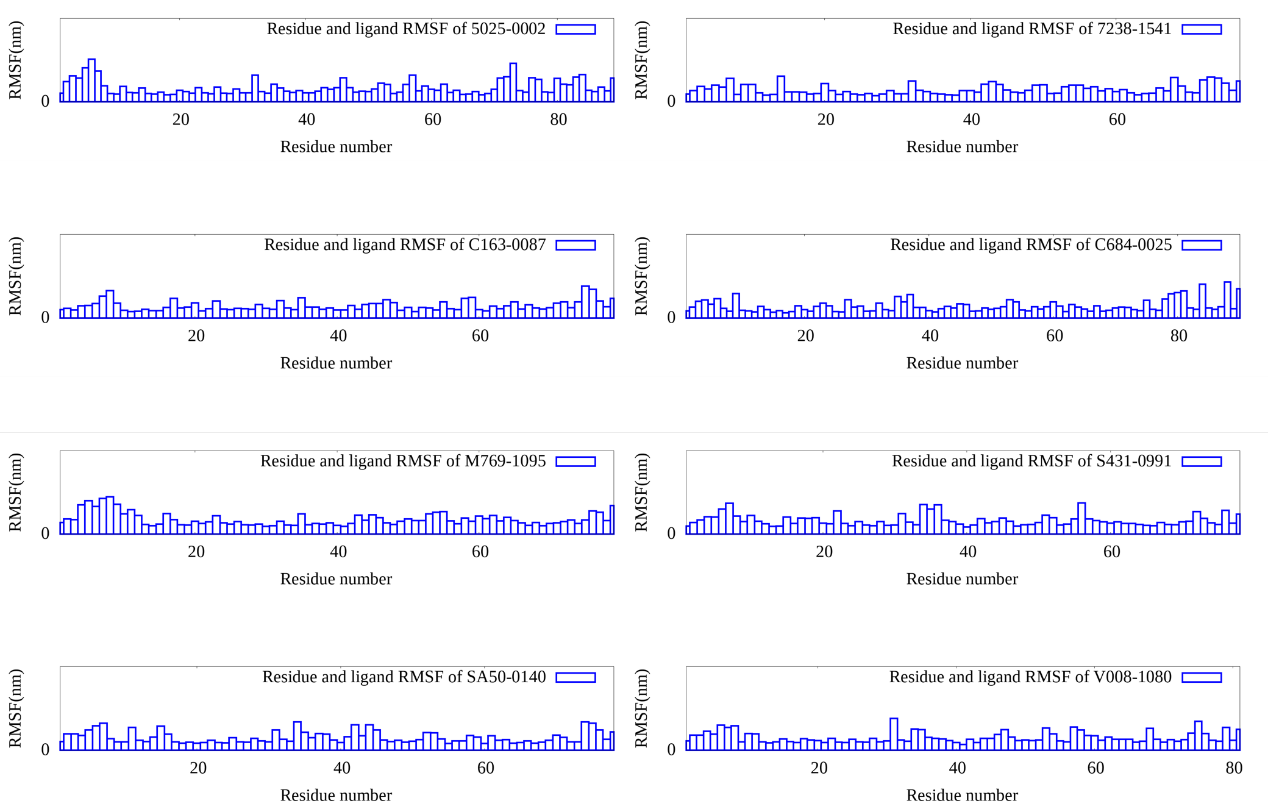
**

**Supplementary Figure S2.** The calculated RMSF of residues and ligand for the selected eight compound-pocket complexes from the pocket MD simulation.

**
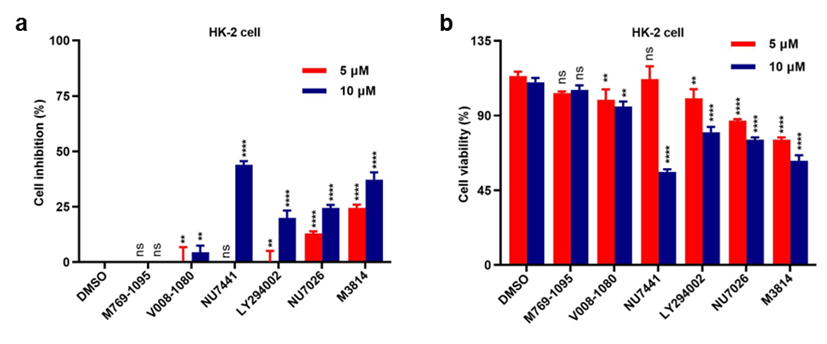
Supplementary Figure S3. DNA-PKcs inhibitors induce cell proliferation inhibition in HK-2 cells. (a and b)** HK-2 cells were treated with M769-1095 and V008-1080 along with representative DNA-PKcs inhibitors (5μM, 10μM) for 72 hours, cell inhibition **(a)** or cell viability **(b)** was analyzed by CCK8 assay. All *P*-values were obtained by comparing to the DMSO group at the same concentration. ns denotes not significant, ** denotes *P* < 0.01, **** denotes *P* < 0.0001.

**
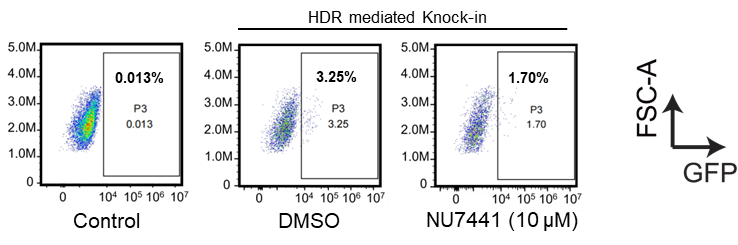
**

**Supplementary Figure S4. Evaluation of HDR-mediated gene knock-in efficiency after treatment with NU7441.**

**
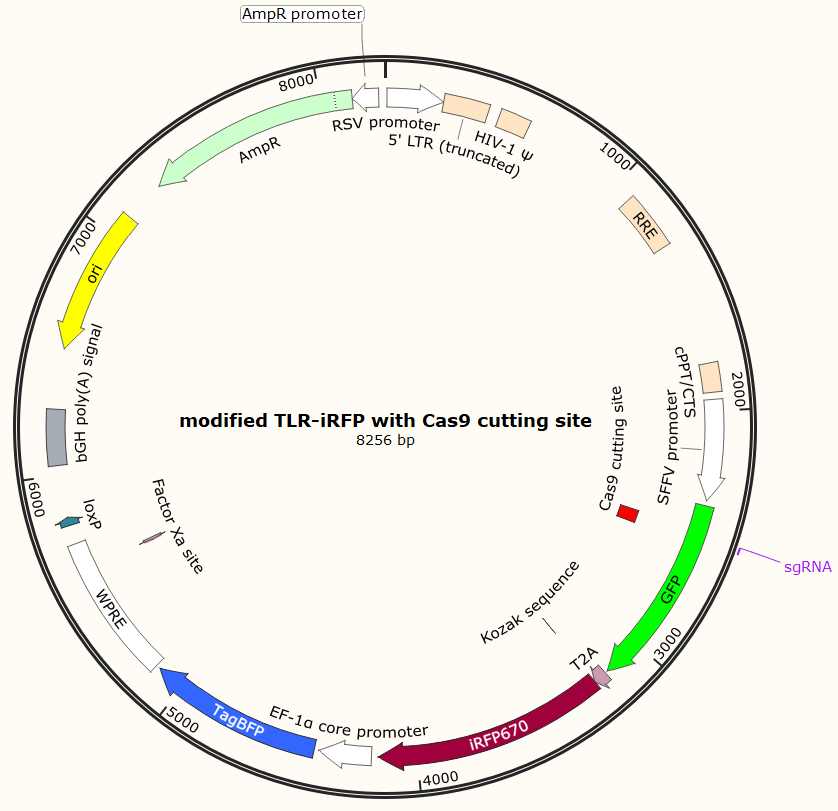
**

**Supplementary Figure S5. Plasmid map of modified Traffic Light Reporter (TLR) with CRISPR/Cas9 cutting site.**


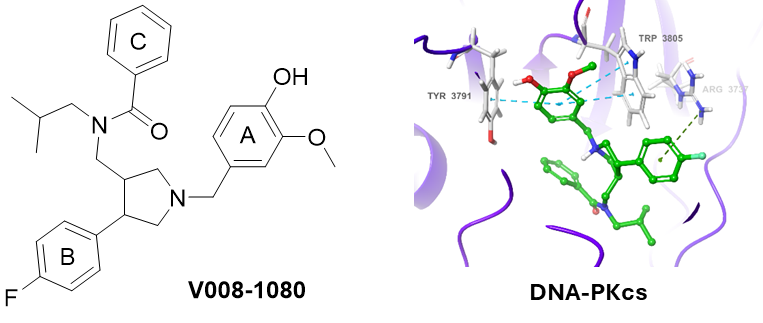


**Supplementary Figure S6. Potential structural modifications of V008-1080.**


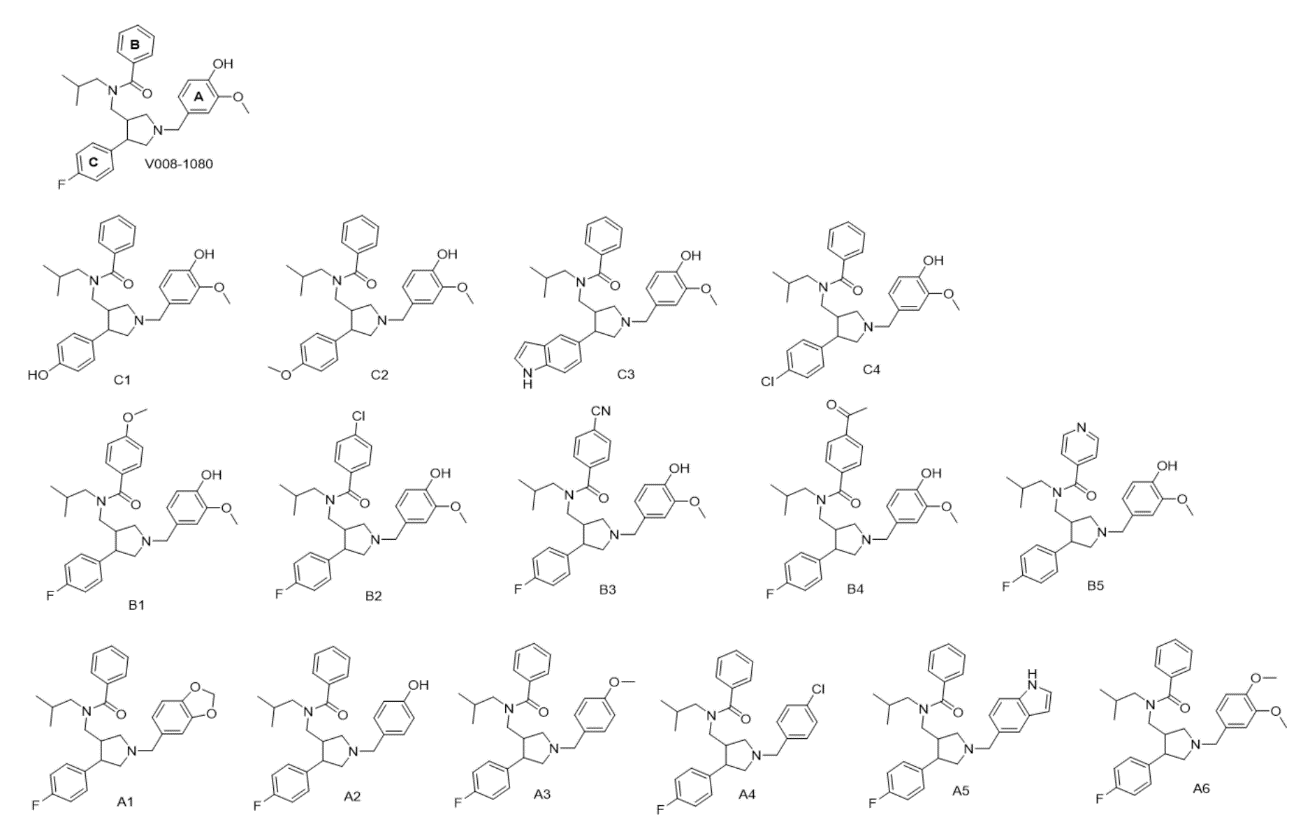


**Supplementary Figure S7. The structure of proposed potential derivates.**

**Supplementary Table S1. Schrödinger docking score for the V008-1080 and designed derivates.**

| **Name** | **Docking score (Kcal/mol)** |
| --- | --- |
| C3 | -9.038 |
| C4 | -8.371 |
| B1 | -7.901 |
| B4 | -7.788 |
| C1 | -7.700 |
| B3 | -7.690 |
| C2 | -7.670 |
| B2 | -7.640 |
| A2 | -7.576 |
| A4 | -7.460 |
| B5 | -7.422 |
| A5 | -7.351 |
| A1 | -7.156 |
| V008-1080 | -7.797 |
| A6 | -6.703 |
| A3 | -6.475 |

**Supplementary Table S2. The Pocket residue ID in MD simulation and its corresponding residue ID in PDB for the 8 final selected candidates.**

| Name | Mapping the Pocket residue ID in MD simulation to residue ID in PDB |
| --- | --- |
| 5025-0002 | 1:3728 2:3729 3:3730 4:3731 5:3732 6:3733 7:3734 8:3735 9:3736 10:3738 10:3736 11:3737 12:3738 13:3748 13:3740 14:3749 15:3750 16:3750 16:3752 17:3751 18:3752 19:3752 19:3754 20:3753 21:3754 22:3755 22:3756 23:3756 24:3757 25:3757 25:3759 26:3758 27:3759 28:3761 28:3760 29:3761 30:3762 31:3764 32:3765 33:3766 34:3768 34:3788 35:3789 36:3790 37:3791 38:3792 39:3793 40:3794 41:3802 41:3796 42:3803 43:3804 44:3805 45:3806 46:3807 47:3808 48:3809 49:3810 50:3811 51:3812 52:3813 53:3814 54:3815 55:3817 55:3921 56:3922 57:3923 58:3924 59:3925 60:3926 61:3927 62:3928 63:3929 64:3930 65:3931 66:3937 66:3933 67:3938 68:3939 69:3940 70:3941 71:3942 72:3943 73:3944 74:3945 75:4018 75:3947 76:4019 77:4020 78:4021 79:4021 79:4023 80:4022 81:4023 82:4024 83:4025 84:4026 85:4027 86:4029 86:4122 87:4123 88:4124 |
| 7238-1541 | 1:3728 2:3729 3:3730 4:3731 5:3732 6:3733 7:3734 8:3735 9:3736 10:3737 11:3738 12:3748 12:3740 13:3749 14:3750 15:3751 16:3752 17:3753 18:3754 19:3755 19:3756 20:3756 21:3757 22:3759 22:3757 23:3758 24:3759 25:3760 25:3761 26:3761 27:3762 28:3764 29:3765 30:3766 31:3768 31:3788 32:3789 33:3790 34:3791 35:3792 36:3793 37:3794 38:3796 38:3802 39:3803 40:3804 41:3805 42:3806 43:3807 44:3808 45:3809 46:3810 47:3811 48:3812 49:3813 50:3814 51:3815 52:3817 52:3921 53:3922 54:3923 55:3924 56:3925 57:3926 58:3927 59:3928 60:3929 61:3930 62:3931 63:3933 63:3937 64:3938 65:3939 66:3940 67:3941 68:3942 69:3943 70:3944 71:3946 71:4021 72:4022 73:4023 74:4024 75:4025 76:4026 |
| C163-0087 | 1:3726 2:3727 3:3728 4:3729 5:3730 6:3731 7:3732 8:3733 9:3734 10:3735 11:3736 12:3736 12:3738 13:3737 14:3738 15:3748 15:3740 16:3749 17:3750 18:3751 19:3752 20:3753 21:3754 22:3755 22:3756 23:3756 24:3757 25:3757 25:3759 26:3758 27:3759 28:3761 28:3760 29:3761 30:3762 31:3764 32:3765 33:3766 34:3788 34:3768 35:3789 36:3790 37:3791 38:3792 39:3793 40:3794 41:3796 41:3801 42:3802 43:3803 44:3804 45:3805 46:3806 47:3807 48:3808 49:3809 50:3810 51:3811 52:3812 53:3813 54:3815 54:3813 55:3814 56:3815 57:3922 57:3817 58:3923 59:3924 60:3925 61:3927 61:3925 62:3926 63:3927 64:3928 65:3929 66:3930 67:3931 68:3932 69:3935 69:3934 70:3936 71:3937 72:3938 73:3939 74:3940 75:3941 76:3942 77:3943 78:3944 |
| C684-0025 | 1:3727 2:3728 3:3729 4:3730 5:3731 6:3732 7:3733 8:3733 8:3735 9:3734 10:3735 11:3736 12:3738 12:3736 13:3737 14:3738 15:3740 15:3750 16:3751 17:3752 18:3752 18:3754 19:3753 20:3754 21:3755 21:3756 22:3756 23:3757 24:3758 25:3759 26:3759 26:3761 27:3760 28:3761 29:3762 30:3763 31:3764 31:3765 32:3765 33:3766 34:3768 34:3788 35:3789 36:3790 37:3791 38:3792 39:3793 40:3794 41:3802 41:3796 42:3803 43:3804 44:3805 45:3806 46:3807 47:3808 48:3810 48:3808 49:3809 50:3810 51:3811 52:3812 53:3813 54:3814 55:3815 56:3921 56:3817 57:3922 58:3923 59:3924 60:3925 61:3926 62:3927 63:3928 64:3929 65:3930 66:3937 66:3932 67:3938 68:3939 69:3940 70:3941 71:3942 72:3943 73:3944 74:4018 74:3946 75:4019 76:4020 77:4022 78:4023 79:4024 80:4025 81:4026 82:4027 83:4120 83:4029 84:4121 85:4122 86:4122 86:4124 87:4123 88:4124 89:4125 |
| M769-1095 | 1:3726 2:3727 3:3728 4:3729 5:3730 6:3731 7:3732 8:3733 9:3734 10:3735 11:3736 12:3737 13:3738 14:3740 14:3748 15:3749 16:3750 17:3751 18:3752 19:3754 19:3752 20:3753 21:3754 22:3755 22:3756 23:3756 24:3757 25:3759 25:3757 26:3758 27:3759 28:3760 28:3761 29:3761 30:3762 31:3764 32:3765 33:3766 34:3768 34:3788 35:3789 36:3790 37:3791 38:3792 39:3793 40:3794 41:3796 41:3802 42:3803 43:3804 44:3805 45:3806 46:3807 47:3808 48:3808 48:3810 49:3809 50:3810 51:3811 52:3812 53:3813 54:3814 55:3815 56:3816 57:3818 57:3921 58:3922 59:3923 60:3924 61:3925 62:3926 63:3927 64:3928 65:3929 66:3930 67:3932 67:3937 68:3938 69:3939 70:3940 71:3941 72:3942 73:3943 74:3944 75:4021 75:3946 76:4022 77:4023 78:4024 |
| S431-0991 | 1:3727 2:3728 3:3729 4:3730 5:3731 6:3732 7:3733 8:3734 9:3735 10:3736 11:3737 12:3738 13:3740 13:3748 14:3749 15:3750 16:3751 17:3752 18:3753 19:3754 20:3755 21:3757 21:3755 22:3756 23:3757 24:3757 24:3759 25:3758 26:3759 27:3761 27:3760 28:3761 29:3762 30:3764 31:3765 32:3766 33:3788 33:3768 34:3789 35:3790 36:3791 37:3792 38:3793 39:3794 40:3796 40:3802 41:3803 42:3804 43:3805 44:3806 45:3807 46:3808 47:3809 48:3810 49:3811 50:3812 51:3813 52:3814 53:3815 54:3817 54:3921 55:3922 56:3923 57:3924 58:3925 59:3926 60:3927 61:3928 62:3929 63:3930 64:3931 65:3932 66:3936 66:3934 67:3937 68:3938 69:3939 70:3940 71:3941 72:3942 73:3943 74:3944 75:3946 75:4022 76:4023 77:4024 |
| SA50-0140 | 1:3728 2:3729 3:3730 4:3731 5:3732 6:3733 7:3734 8:3735 9:3736 10:3736 10:3738 11:3737 12:3738 13:3748 13:3740 14:3749 15:3750 16:3751 17:3752 18:3752 18:3754 19:3753 20:3754 21:3756 21:3755 22:3756 23:3757 24:3757 24:3759 25:3758 26:3759 27:3761 27:3760 28:3761 29:3762 30:3764 31:3765 32:3766 33:3768 33:3788 34:3789 35:3790 36:3791 37:3792 38:3793 39:3794 40:3796 40:3802 41:3803 42:3804 43:3805 44:3806 45:3807 46:3808 47:3808 47:3810 48:3809 49:3810 50:3811 51:3812 52:3813 53:3814 54:3815 55:3922 55:3817 56:3923 57:3924 58:3925 59:3926 60:3927 61:3928 62:3929 63:3930 64:3931 65:3933 65:3937 66:3938 67:3939 68:3940 69:3941 70:3942 71:3943 72:3944 73:3946 73:4022 74:4023 75:4024 76:4025 77:4026 |
| V008-1080 | 1:3727 2:3728 3:3729 4:3730 5:3731 6:3732 7:3733 8:3734 9:3735 10:3736 11:3737 12:3738 13:3740 13:3748 14:3749 15:3750 16:3751 17:3752 18:3753 19:3754 20:3755 21:3757 21:3755 22:3756 23:3757 24:3759 24:3757 25:3758 26:3759 27:3760 27:3761 28:3761 29:3762 30:3764 31:3765 32:3766 33:3768 33:3788 34:3789 35:3790 36:3791 37:3792 38:3793 39:3794 40:3800 40:3796 41:3801 42:3802 43:3803 44:3804 45:3805 46:3806 47:3807 48:3808 49:3809 50:3810 51:3811 52:3812 53:3813 54:3814 55:3815 56:3817 56:3922 57:3923 58:3924 59:3925 60:3926 61:3927 62:3928 63:3929 64:3930 65:3931 66:3932 67:3932 67:3934 68:3933 69:3934 70:3937 70:3936 71:3938 72:3939 73:3940 74:3941 75:3942 76:3943 77:3944 78:3946 78:4022 79:4023 80:4024 |
